# Shoulder kinematics plus contextual target information enable control of multiple distal joints of a simulated prosthetic arm and hand

**DOI:** 10.1186/s12984-020-00793-0

**Published:** 2021-01-06

**Authors:** Sébastien Mick, Effie Segas, Lucas Dure, Christophe Halgand, Jenny Benois-Pineau, Gerald E. Loeb, Daniel Cattaert, Aymar de Rugy

**Affiliations:** 1 Institut de Neurosciences Cognitives et Intégratives d’Aquitaine, UMR 5287, CNRS and Univ. Bordeaux, 146 rue Léo Saignat, 33076 Bordeaux, France; 2 Laboratoire Bordelais de Recherche en Informatique, UMR 5800, CNRS, Univ. Bordeaux and Bordeaux INP, 351 cours de la Libération, 33405 Talence, France; 3 Department of Biomedical Engineering, Univ. Southern California, 1042 Downey Way, Los Angeles, CA 90089 USA; 4 Centre for Sensorimotor Performance, School of Human Movement and Nutrition Sciences, Univ. Queensland, Blair Drive, Brisbane, QLD 4059 Australia

**Keywords:** Arm prosthesis, Movement-based control, Joint angle prediction

## Abstract

**Background:**

Prosthetic restoration of reach and grasp function after a trans-humeral amputation requires control of multiple distal degrees of freedom in elbow, wrist and fingers. However, such a high level of amputation reduces the amount of available myoelectric and kinematic information from the residual limb.

**Methods:**

To overcome these limits, we added contextual information about the target’s location and orientation such as can now be extracted from gaze tracking by computer vision tools. For the task of picking and placing a bottle in various positions and orientations in a 3D virtual scene, we trained artificial neural networks to predict postures of an intact subject’s elbow, forearm and wrist (4 degrees of freedom) either solely from shoulder kinematics or with additional knowledge of the movement goal. Subjects then performed the same tasks in the virtual scene with distal joints predicted from the context-aware network.

**Results:**

Average movement times of 1.22s were only slightly longer than the naturally controlled movements (0.82 s). When using a kinematic-only network, movement times were much longer (2.31s) and compensatory movements from trunk and shoulder were much larger. Integrating contextual information also gave rise to motor synergies closer to natural joint coordination.

**Conclusions:**

Although notable challenges remain before applying the proposed control scheme to a real-world prosthesis, our study shows that adding contextual information to command signals greatly improves prediction of distal joint angles for prosthetic control.

## Background

Myoelectric prostheses in which movements of the prosthetic joints are controlled by the activity of remaining muscles face a fundamental dimensionality problem: the higher the amputation, the more artificial degrees of freedom to control with fewer remaining muscles. Despite progress in myoelectric signal analysis [1, 2] and in muscle reinnervation surgery that aim at recovering original control signals [3, 4], myoelectric signals are inherently noisy and hard to process for natural movement control. This challenge increases as prosthetic hands with more anthropomorphic articulations become available (e.g [5]).

To overcome these limitations, alternative controls have been explored, particularly using the kinematics of remaining proximal joints, which are far less subject to artefacts and difficulties of interpretation. Several studies have demonstrated that known regularities in joint coordination during reach and grasp movement with the arm [6–8] could be exploited to reconstruct missing distal joints from that of remaining proximal ones for prosthesis control [9–18]. This approach faces a dimensionality problem similar to that associated with myoelectric control, because higher amputation still requires more distal joints to be controlled by fewer proximal degrees of freedom. In this context, it is revealing that most of those attempts have been restricted to the sole control of an artificial elbow on the bases of actual shoulder movements [9, 10, 15–18]. This is in principle sufficient to enable people with trans-humeral amputation to reach various positions in space, but it is not good enough for them to correctly orient their prosthetic hand to grasp oriented objects using the additional degrees of freedom (DoF) that are now available in some prosthetic limbs.

The requirement for hand orientation was partly addressed by including prediction of forearm pronation-supination to reach, in a large 3D workspace, cylindrical objects with various tilts in the frontal plane [12]. This was extended to control hand closure to grasp variously oriented objects [13] but accurate performance required 3–5 training sessions of 15–30 min each and reach-and-grasp tasks took about twice the time compared to virtual reaches using complete arm and hand kinematics. Kinematic control of the two degrees of freedom in the wrist (flexion-extension and radial-ulnar deviations) has not been demonstrated and would be necessary to efficiently grasp randomly oriented objects without requiring large compensatory movements by the trunk and shoulder to bring the hand to the desired orientation.

We have investigated a solution for the dimensionality problem by adding contextual information about the movement goal to predict the desired posture of more distal joints of a prosthetic limb. The present experiment aims at assessing the benefit of such an approach by comparing a control based only on shoulder kinematics with a control based on shoulder kinematics plus coordinates expressing the position and orientation of the target to reach. Given recent progress in computer vision augmented with gaze information, it is now feasible to identify objects of interest [19–21] and to identify the position and orientation of the target object with respect to the subject. This is relatively easy with simplified real-world scenes containing uniformly colored objects of pre-defined geometric shapes [19, 20]. More recently, our research group developed approaches that achieved mean accuracy of $75 \pm 3.3\%$ for every-day objects in very cluttered natural environments such as kitchens [21].

In the experiment reported here, we collected complete postural data from a single practice session of picking and placing a bottle in various positions and orientations in a 3D virtual environment. These data were used to train neural networks to reconstruct postures of the elbow, forearm and wrist, either solely from shoulder joint angles (context-unaware network), or with additional knowledge of location and orientation of the movement goal (context-aware network). Our results show that subjects using the context-aware network achieved close to natural performance and moderate compensatory movements without training. In contrast, performance deteriorated significantly and required substantial compensatory movements when using the context-unaware network. It is worth noting that the present experiment assessed the benefit of one specific set of contextual information, picked to be the most relevant in the framework of a specific task. However, one can reasonably expect similar outcomes in other cases, even when the choice of coordinates acting as contextual information is suboptimal. Following a similar approach, recent reports [22, 23] have investigated how various forms of knowledge about the user’s movement intention or goal can improve the control of a prosthetic or assistive robotic arm.

The present experiment employs a virtual reality (VR) environment to emulate the control of a trans-humeral prosthesis with able-bodied subjects. Despite its notable differences compared to a genuine prosthesis worn on a disabled limb, this type of experimental approach has proven useful for research on arm prosthetics [13, 14, 18, 22, 24, 25], especially for exploring and evaluating prototype control schemes. The goal-related additional data were available through the virtual environment’s simulation engine, which can directly provide the absolute location of every object involved in the simulation, including the goal itself. Obviously, this kind of “omniscient” point of view would not be available for a clinical system usable outside the lab. Instead, the computer-vision-based tools previously described would provide data from an egocentric point of view. In this sense, the present experiment should be seen as a proof of concept focused on the benefit of such data for prosthesis control in the first place. The question of how to acquire such goal-related data is discussed along with other technology needed to achieve useful function with a clinical prosthesis.

## Methods

### Participants

The study was conducted on a set of ten naive right-handed subjects (six male), aged 23–48 (mean 29.6; SD 7.7) with normal or corrected-to-normal vision. All subjects were able-bodied and none of them suffered from any mental or motor disorder that could affect their ability to perform the task. The experiment duration ranged from 45 to 75 min, and no subject reported fatigue at the end of the experiment.

### Apparatus

**Fig. 1:**
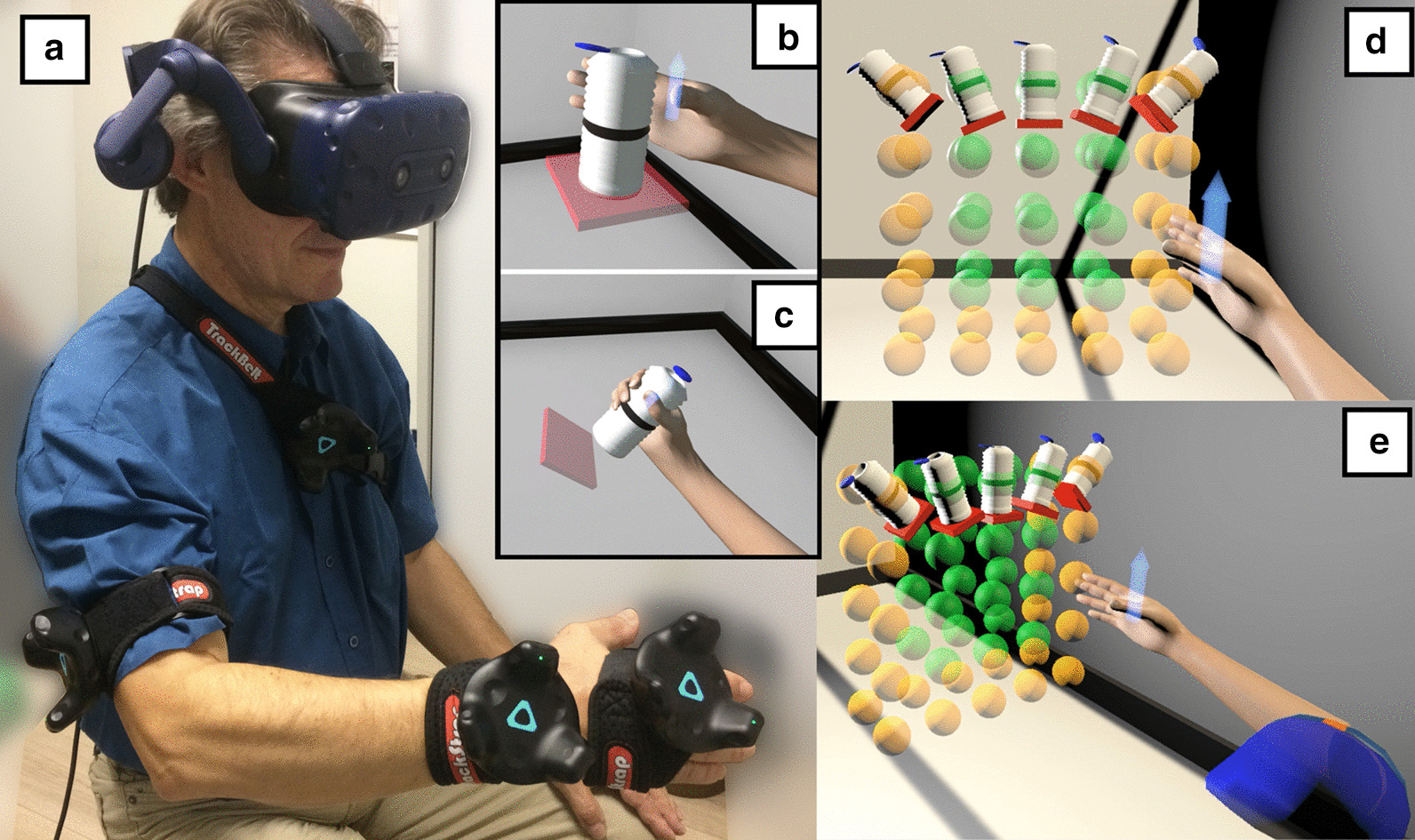
Experimental setup. **a** Subject equipped with the four motion trackers and headset. Subject about to pick (**b**) and place (**c**) the bottle. Corresponding virtual arm, next to the layout of the two target sets in the virtual scene, from an egocentric (**d**) or side (**e**) point of view. The whole target set includes positions of all spheres (yellow and green), and the target subset includes only the positions of green spheres. The five possible target orientations are shown on the top row

Subjects were seated on a chair and wore a virtual reality headset (Vive^TM^ Pro, HTC Corporation) adjusted by the experimenter to fit their head firmly and comfortably. Four motion trackers (Vive^TM^ Tracker, HTC Corporation) were attached to the subject’s body using armbands or straps, so that the trunk as well as each arm segment (upper arm, forearm and hand) had a dedicated tracker attached to it (Fig. 1a). Each tracker as well as the headset provided measurements of their 3D position and 3D orientation relative to the virtual environment’s reference frame. The two beacons that receive tracking signals from the headset and trackers were placed and calibrated so that the virtual environment’s workspace was centered on the chair. Additionally, the virtual environment was scaled to match real-world dimensions and its ground plane was set at the same height as the actual floor.

Using SteamVR (Valve Corporation) as middleware, the headset and trackers’ 3D positions and orientations were tracked at 90 Hz sampling rate, while the virtual environment was displayed synchronously to the subject at 90 Hz refresh rate. The virtual scene’s contents and interaction with the subject were managed by the Unity simulation engine (Unity Technologies).

### Virtual arm calibration

The scene included a virtual arm, whose skeleton consisted of three rigid segments (upper arm, forearm and hand) linked to each other by spherical joints. After the subject was equipped with the headset and trackers, a calibration procedure was carried out to “link” this virtual arm to the trackers, so that its motion mimicked the subject’s right arm motion from the shoulder to the wrist:

1. The method described in [26] was used to estimate each of the subject’s joint centers’ locations relative to its parent tracker: shoulder relative to the trunk tracker, elbow relative to the upper arm tracker, and wrist relative to the forearm tracker.
2. Motion data was collected as ten-second recordings during which the subject was asked to perform slow movements using all of their arm’s degrees of freedom: shoulder flexion-extension, abduction-adduction and humeral rotation, elbow flexion, forearm pronation-supination, wrist flexion-extension and radial-ulnar deviation. The estimated joint centers were displayed as red spheres in the virtual scene so that the experimenter could check the accuracy of the method’s output. During this step, the trackers’ silhouettes were also displayed and worked as anatomical landmarks to compare the estimated locations with those of the actual joints. In the event of an insufficiently accurate output, the first step was redone until a satisfying estimation was obtained.
3. The virtual arm’s skeleton was locked in a reference posture, scaled to match the dimensions of the subject’s arm and placed so that its shoulder coincided with the subject’s estimated shoulder location.
4. The subject was asked to put their arm in the same posture by overlaying the red spheres, which represented estimated joint centers, on the virtual arm’s joints. When an appropriate overlaying was found, the calibration procedure ended: the red spheres and trackers’ silhouettes disappeared and the virtual arm was unlocked to be able to mimic the subject’s arm motion.

### Task

During the experiment, the subject was asked to use the virtual arm to reach and pick a cylindrical bottle at a given location in the virtual scene (Fig. 1b), then place it at another location (Fig. 1c). The goal was to complete a fixed sequence of tasks in the shortest total time. Here, the word “task” will be used to refer to only one part of this process: either the bottle-picking or the bottle-placing. Given that subjects had no control over the virtual fingers, the task did not involve closing or opening the hand to grasp or release the bottle. Instead, the task was achieved by holding the virtual hand inside a target zone for one second. In the six-dimensional space of hand locations (3D position $\times$ 3D orientation), this target zone corresponded to the region containing all hand locations with the same position and orientation as the target, within a margin defined by a spatial tolerance and an angular tolerance. The former defined the maximum distance between the hand’s center and the target’s center, while the latter defined the maximum angle between the hand’s axis and the target’s axis.

During a bottle-picking task, the virtual hand was empty and the target corresponded to the bottle itself. Accordingly, the target’s center was placed at the middle of the bottle’s height and its axis was the bottle’s orientation in the frontal plane. During a bottle-placing task, the hand was holding the bottle and the target corresponded to a small rectangular plate. Accordingly, the target’s axis was perpendicular to the plate’s plane and its center was placed so that a correct hand positioning would bring the bottom of the bottle against the plate. This adjustment was made so that the task’s instruction i.e. “place the bottle on the plate” would remain intuitive to the subject. Additionally, a semi-transparent arrow was attached to the virtual hand (see Fig. 1) to indicate the hand’s center (base of the arrow) and axis (direction of the arrow). When the target zone was entered, the bottle turned red to indicate that a correct hand location was reached. Collisions were ignored; the virtual hand could go through the bottle or plate during a trial without affecting the target’s location.

The subject was allowed a maximum of 15 s to complete each task. Failure to complete the task within this allotted time triggered the end of the current trial, indicated by a short audio cue. The target state of the hand i.e. either open and empty, or closed and holding the bottle, was automatically set to completion at the end of each trial regardless of success. The subject’s trunk was not restrained, but the subject was instructed to keep it against the backrest of the chair unless trunk motion was required to perform the task.

### Target sets

Each target i.e. each bottle-picking or bottle-placing location used in this experiment was defined by four spatial parameters. The first three parameters were the Cartesian coordinates of the target’s center in the virtual scene. The last parameter was the angle by which the corresponding object (either bottle or rectangular plate) was rotated in the frontal plane only, selected from − 45$^{\circ }$; − 22.5$^{\circ }$; 0$^{\circ }$; 22.5$^{\circ }$; 45$^{\circ }$ (positive = counterclockwise). Possible positions for target centers were distributed along a 3D orthogonal grid with a regular unit spacing of 8 cm along the three dimensions. The grid was five-units high (top to bottom), five-units wide (left to right) and two-units deep (front to back) for a total of fifty positions (Fig. 1d, e). With respect to the subject, the center of the grid was roughly aligned with the shoulder. As a result, the target grid spanned a relatively small portion of the subject’s overall peripersonal space corresponding to comfortably unconstrained reaches. After the calibration procedure, an estimation of the subject’s arm length allowed to check that all positions in the grid were reachable without putting the arm in an extreme or uncomfortable posture.

The experiment made use of two distinct target sets. One is referred to as the whole target set and included all the combinations of the fifty positions with the five orientations, for a total of 250 targets. The other is referred to as the target subset and included the combinations of only twenty-four positions with the five orientations, for a total of 120 targets. These twenty-four positions were obtained by excluding the leftmost, rightmost and lowest positions in the grid as discussed below (green spheres in Fig. 1d, e).

For each subject, two target orders were generated from these two sets. A target order defines a sequence alternating between picking locations (bottles) and placing locations (rectangular plates). Following a target order, tasks were performed one after another without subjects having to go back to an initial state. In this way, the virtual hand’s location at the end of a task was its location at the beginning of the next trial. At the end of a placing task, the bottle and the plate were instantaneously moved to the next target locations, so that the next pick-and-place process could begin immediately afterwards.

The order generation process consisted of randomly drawing targets from a given set in a way that prevented two consecutive targets from being located at neighboring positions. The process ended when no appropriate target could be drawn to follow the last-picked target. As a consequence, target orders could have different sizes depending on the subjects. However, a rule was implemented so that orders included at least 200 targets if generated from the whole set, and 100 targets if generated from the subset. Performance of various neural networks was compared for the same target order in a given subject in order to facilitate comparison of performance metrics.

### Protocol

The experimental protocol was divided into five distinct phases, each comprising several trials of the task. Within a phase, trials were grouped in blocks of fifty tasks consisting of twenty-five repetitions of the pick-and-place process. Short pauses ($< 1\,\hbox {min}$) were allowed between blocks so that subjects could rest and relax their arm if needed. Additionally, the completion time of the last block was shown during a pause, and subjects were encouraged to complete the next block within a shorter time, as long as this was not at the cost of accuracy or task success.

#### Familiarization

The first phase of the experiment was a familiarization phase during which subjects performed a single block of fifty trials with the virtual arm mimicking their right arm’s motion. No data were recorded from these trials. This phase’s targets followed the first fifty items of the order generated from the whole target set.

The 15 s time limit condition was disabled so that the subject would not be interrupted during a familiarization trial and could therefore explore the apparatus freely. In the case of a subject getting stuck on a given trial, the experimenter could manually skip to the next so that the familiarization could go on.

#### Initial data acquisition

The second phase of the experiment was dedicated to the acquisition of motion tracking data in the framework of the task. This phase’s targets followed the order generated from the whole target set, for a total of at least 200 trials. These trials were performed with the virtual arm mimicking the motion of the subject’s right arm. The time limit condition was enabled again, and the spatial and angular tolerances were set at 2 cm and 5$^{\circ }$, respectively, based on preliminary experiments.

Motion tracking data was recorded throughout this phase, in the form of 3D positions of estimated joint centers and 3D orientations of the virtual arm’s segments. Recordings also included target positions and orientations along time, as well as the beginning and end of each trial.

#### Neural network training

The data acquired during the second phase was filtered to include only samples during which the virtual hand’s location was inside the target zone. Then, these samples were processed to build a training dataset.

Joint angles were computed based on the recorded segment orientations. The kinematic model of the arm underlying the computation of these angles comprised three segments and seven DoFs: three at shoulder level (flexion-extension, abduction-adduction and humeral rotation), one at elbow level (flexion-extension), one at forearm level (pronation-supination), and two at wrist level (flexion-extension and radial-ulnar deviation).

Additionally, *contextual information* was computed from the position of the shoulder estimated by the earth-based motion tracking and the location and orientation of the target already known to the simulation system. It consisted of the 3-dimensional vector from the estimated shoulder center to the target’s center plus the target’s rotation angle. In this way, a sample of target-related contextual information is given as a quadruplet including 3 spatial coordinates and 1 angular coordinate, expressed in an egocentric frame attached to the subject’s shoulder. For a wearable prosthetic control system, an earth-based reference frame would not be available but similar contextual information could be computed using other technology described in the Discussion.

**Fig. 2:**
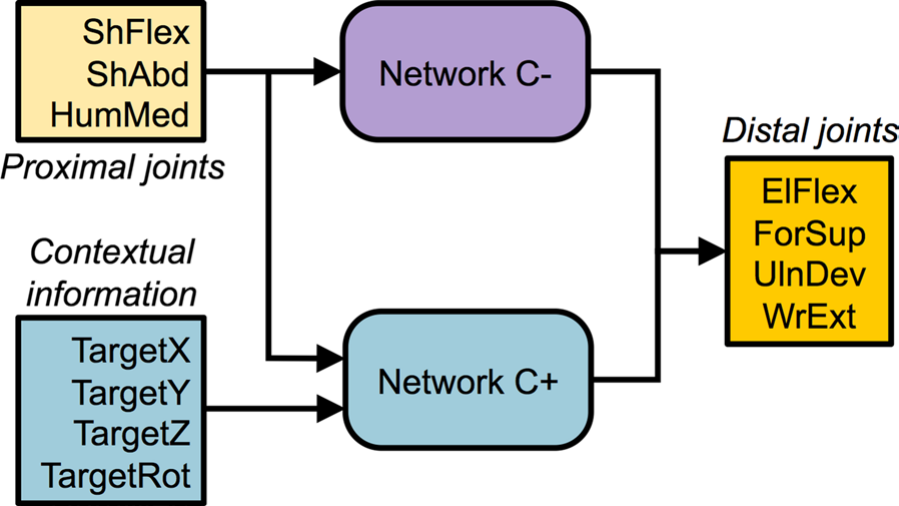
Diagram of the neural networks’ inputs and outputs. *ShFlex* shoulder flexion, *ShAbd* shoulder abduction, *HumMed* Humeral lateral rotation, *ElFlex* elbow flexion, *ForSup* forearm supination, *UlnDev* ulnar deviation, *WrExt* wrist extension

The dataset of joint angles and contextual information was then fed to two artificial neural networks to train them to predict the four distal joint angles (elbow, forearm and wrist DoFs). These networks shared the same output but worked with different inputs (Fig. 2). The first network only received the three shoulder angles as input whereas the second network received these angles as well as the contextual information (three Cartesian coordinates and one angle). Accordingly, the former is referred to as the context-unaware network (labeled C−) while the latter is referred to as the context-aware network (labeled C+).

Except for their input layer (size 3 and 7, respectively), both networks shared the same structure: two dense layers of 256 neurons each, a dropout layer with a drop fraction of 0.5, a dense layer of 64 neurons, and an output layer of 4 neurons. We employed Tensorflow [27] as the backend and Keras [28] as the programming interface to implement and train these networks.

#### Test

During the third and fourth phases of the experiment, the virtual shoulder kept mimicking the subject’s shoulder motion but the virtual forearm and hand stopped following the corresponding trackers. Instead, the virtual elbow, forearm and wrist were driven based on online predictions from one of the two networks. This hybrid control strategy was designed to emulate the behavior of a trans-humeral prosthesis, where the wearer’s residual limb motion is combined with the prosthesis’s actuation to perform a movement with the whole arm. As a consequence, the virtual arm’s behavior did not necessarily match the subject’s real arm motion, the latter being invisible to the subject inside the virtual environment. Subjects were advised of this condition and instructed to achieve the best performance with whatever strategy they could devise. The criteria for a successful trial were relaxed to 4 cm and 10$^{\circ }$.

**Fig. 3:**
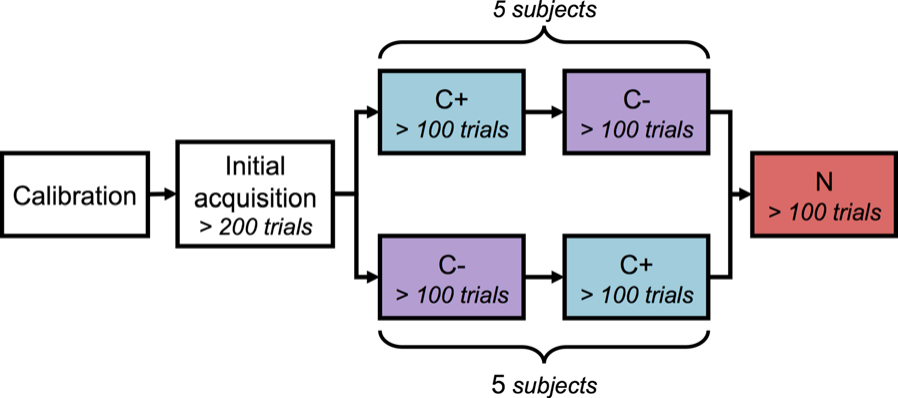
Experimental design. Subjects are divided in two groups of same size, each corresponding to a different order of conditions during test phases. *C+* context-aware network, *C−* context-unaware networ, *N* natural arm motion

These two phases are referred to as test phases, as they were dedicated to the evaluation of performance in the framework of the task, in order to compare the quality of arm control achieved with each network. Each of the test phases was performed with joint angle predictions from one of the two networks, either C+ or C−. The order was counterbalanced over subjects so that half of them performed their first test phase with network C+ while the other half began with network C− (Fig. 3).

Test phase targets followed the order generated from the target subset, for a total of at least 100 trials (see “Target sets” section). In this way, the network’s prediction corresponded to targets located on the inside of the portion of space that was covered by the training data collected throughout the first phase. This was designed to avoid the detrimental effects of potentially poorer prediction accuracy for input data on the edge of the whole target set. Indeed, preliminary pilot testing revealed that hybrid control deteriorated substantially for some targets located on and beyond the boundaries of the space covered in the second phase used to train the networks.

#### Baseline

During the fifth phase of the experiment, the virtual arm went back under its original control i.e. mimicking the subject’s complete arm motion. This phase is referred to as the baseline phase, as it was dedicated to the evaluation of performance achieved with natural arm control in the framework of the task. For the sake of comparison with test phases, targets also followed the order generated from the target subset and the spatial and angular tolerances were kept to their test values of 4 cm and 10$^{\circ }$, respectively. Instructions and recordings remained the same as before.

### Data reduction and analysis

#### Offline assessment of network performance

The quality of joint angle prediction achieved by the trained networks was assessed offline in order to evaluate how well each network was able to fulfill its function. As “ground truth”, this assessment made use of motion tracking data from the initial data acquisition and baseline phase, during which the virtual arm followed the real arm’s behavior.

For each subject, the assessment made use of two datasets that were fed to both the context-aware and context-unaware networks. The first dataset was the training dataset built from the recording of the second phase; the second was built from the recording of the fifth phase (baseline) following the same method, including only samples during which the virtual hand’s location was inside the target zone. For a given dataset, the network’s output was the array of four predicted distal joint angles, with as many samples as the dataset. The quality of prediction was evaluated by computing the root mean square error (RMSE) between this array of predicted angles and the corresponding actual angles. In this way, a value of RMSE was computed for each combination of subject, network (C+ or C−) and type of dataset (training or baseline).

In order to compare how well subjects performed the task depending on the virtual arm’s control, the rest of the analysis considered three experimental conditions:

- C+: Hybrid control with predictions from the context-aware network.
- C−: Hybrid control with predictions from the context-unaware network.
- N: Natural control, mimicking the subject’s arm motion.

The corresponding dataset included the recordings from the test and baseline phases, for a total of thirty recorded phases (ten subjects $\times$ three conditions), from which 4062 trials were processed. Over all conditions, the success rate was found to be above 90% for twenty-eight out of these thirty recorded phases, with only one phase yielding a success rate below 85%. This result motivated the design of two quantitative metrics to evaluate the quality of control by addressing various dimensions of motor performance.

#### Shoulder position spread volume (SV)

Based on the recorded 3D positions of the estimated shoulder’s center, this metric evaluates how scattered the shoulder’s position was in the virtual environment in order to assess the amount of compensatory trunk motion performed by the subject. To generate a single performance metric, we computed the volume of a shape representative of the space covered by all the shoulder positions. This shape was an ellipsoid whose dimensions were proportional to the shoulder position’s variability along each direction of space, and included at least 90% of the recorded positions. In this way, a higher volume indicates more scattered shoulder positions. Therefore, we assume that high values of SV are associated with wide and/or frequent compensatory motion performed with the trunk or scapula to move the shoulder’s center. Conversely, we expect that low values of SV only reflect the scapular motion that is part of the upper-body coordinations naturally involved in reaching. For the sake of clarity, the wording “shoulder motion” will refer here to the motion of the shoulder center regardless of the upper body joints actually involved e.g. spine and gleno-scapulo-humeral complex.

This metric assigns one value to each phase by considering the recorded shoulder trajectory in its entirety. For a given recorded phase, we computed the covariance matrix $Cov_{sh}$ of the estimated shoulder’s 3D coordinates over all samples. Being a symmetric matrix, $Cov_{sh}$ is diagonalizable and its eigenvectors form an orthogonal matrix. The corresponding change of basis is an isometric transformation into a reference frame where shoulder coordinates “co-variate” purely along the Cartesian axes. In this alternative reference frame, we computed the volume of the ellipsoid centered on the mean shoulder position and whose semi-axes have each a length equal to three times the standard deviation along this axis. As a reference, assuming 3D shoulder positions followed a multivariate normal distribution, such an ellipsoid would contain approximately 97% of all positions. Given that the alternative frame is obtained through an isometric transformation, the volume computed in this frame is equal to the volume in the original reference frame.

#### Approach speed (AS)

This metric evaluates how fast a subject managed to bring the virtual hand inside the target zone. It assigns one value to each trial where the target zone was entered at least once, which occurred on 3918 trials out of the 4062 trials (> 96%). For any such trial, we were able to compute the approach time (AT) i.e. time elapsed since the beginning of the trial until the first entry of the virtual hand in the target zone. However, given that trials started and ended at various locations in the virtual scene, this time measurement is not appropriate as a target-independent metric. Instead we used the mean *approach speed*, which is defined as the ratio of the distance to the target’s center by the approach time. A lower approach speed means that the control was less capable of bringing the virtual hand inside the target zone, whereas a higher approach speed indicates that the subject could use the control efficiently to drive the virtual hand.

#### Variability of average reaching postures

In order to investigate how the virtual arm behaved while the subject was performing the task, we focused on the variability of its postures when dwelling in the target zone. A lower variability of these postures would mean that the control involved a more homogeneous strategy for bringing the virtual hand at the multiple target locations. Conversely, a higher variability would reveal that the subject had to resort to notably different arm postures to reach the targets, possibly indicating compensatory motion from proximal joints.

Similarly to approach speed, this analysis is only applicable for trials during which the target zone was entered at least once. We focused specifically on the last period during which the virtual hand stayed inside the target zone, corresponding to the one-second holding period when the task was successful. For each sample during this period, the virtual arm’s joint angles were computed offline based on the recorded orientations of each segment. Additionally, for trials from conditions C+ and C−, postures that would have been displayed by the virtual arm should it had kept mimicking the subjects’ whole arm motion were computed offline based on the tracker’s orientations. Then, these joint angles were averaged over all samples of the holding period, yielding a single posture per trial.

This processing resulted in five groups of seven-angle arm postures. Three groups correspond to the virtual arm’s actual behavior in conditions N, C+ and C−. The two remaining groups correspond to the virtual arm’s “simulated” behavior if it had kept mimicking the natural arm’s motion during trials in conditions C+ and C−. These groups are labeled respectively MC+ and MC− to indicate that they are based on data from hybrid control conditions but represent a hypothetical mimicking arm. Because the virtual upper arm followed the subject’s shoulder motion in all conditions, shoulder angles are identical for groups C+ and MC+ as well as for groups C− and MC−. For each phase, we computed the standard deviations (SD) of joint angles from the average reaching postures, resulting in a ten-value sample for each combination of DoF and group. Then we sorted these samples to form two sets with one group from each condition:

- Set corresponding to the virtual arm’s actual behavior, that is: {N, C+, C−}.
- Set corresponding to the mimicked natural motion, that is: {N, MC+, MC−}.

#### Analysis of joint angle synergies with PCA

In addition to the study of joint angle variability, we investigated the synergies underlying the virtual arm’s joint angles when the target was reached. By comparing these synergies, we assessed how similar each hybrid control strategy was to natural arm motion in terms of joint coordinations. This analysis was achieved by conducting principal components analyses (PCA) on the average reaching postures previously computed. For each set of these postures sorted by subject and group, a PCA was carried out and yielded seven principal components (PC), in the form of vectors in the 7-D space of joint angles. As a first step in the analysis of PCA outputs, we compared the cumulated ratios of explained variance from one to seven PCs, between the five groups (C+, C−, MC+, MC−, N). In this context, the amount of variance explained by a given number of PCs corresponds to the prominence of joint angle synergies in the actual or virtual arm’s motion.

The second step consisted in assessing the similarity between joint angle synergies from different groups. This was achieved by evaluating the geometric proximity between subspaces generated by PC vectors corresponding to two given PCAs. Indeed, for a given $n < 7$, the first *n* PCs extracted by a PCA span a subspace within the 7-D joint space. As all PCs are orthogonal with each other by definition, this subspace is *n*-dimensional. In order to measure a distance between two such subspaces, we employed the method described in [8], which finds the minimal angle that rotates one subspace into the other. A smaller angle means a closer proximity between subspaces: 0$^{\circ }$ represents identity whereas 90$^{\circ }$ represents orthogonality. It is worth noting that the use of this method is made possible by the fact that all values involved in posture data are joint angles measured in degrees. As a result, all seven dimensions of the joint space are equivalent in terms of scale, and their magnitude is immediately comparable.

We identified two types of comparison between groups that are relevant for our analysis:

- Between hybrid control and natural control in baseline phase i.e. C+ versus N and C− versus N.
- Between hybrid control and mimicked real arm motion produced in the same test phase i.e. C+ versus MC+ and C− versus MC−.

#### Statistical testing

We carried out statistical testing on the values of RMSE computed for the offline assessment of network performance. For each type of dataset (training or baseline), we compared the quality of prediction achieved by networks C+ and C− using paired T-tests.

Tests were also conducted on the results from the two quantitative metrics to detect significant differences between conditions. Even though approach time was not considered a dependent variable, this quantity was summarily analyzed to provide reference data. In order for each subject to have a similar weight despite slight variations in the number of valid trials, we sorted values of AS and AT by subject and condition, then averaged them over trials. In this way, we obtained samples of ten values (one per subject) for each combination of metric and condition.

We performed either one-way ANOVAs or Kruskal–Wallis tests depending on whether the parametric hypotheses (i.e. normality of distributions and homoscedasticity of samples) were verified. In all the cases where these tests indicated significant differences, post hoc tests (either paired T-tests or Wilcoxon tests) were carried out to identify the pairs of conditions presenting such differences, applying the appropriate Bonferroni correction.

Following the same testing method, we analyzed the SDs of joint angles from the average reaching postures. For each DoF, we compared the different groups based on the sets previously defined: {C+, C−, N} and {MC+, MC−, N}. In the case of a shoulder DoF, only one set was analyzed, considering that both sets are identical.

Data processing and statistical testing were carried out with custom software developed in Python using several packages from the SciPy ecosystem [29]. The significance threshold was set at $\alpha$ = 0.05. When Bonferroni correction was applied, the threshold was adjusted to $\alpha _{corr}$ = 0.05/3 = 0. 0167.

## Results

### Offline network performance

With both types of dataset (training and baseline), the offline error between actual and predicted joint angles was significantly lower for network C+ than for network C− ($\hbox {p} < 0.00001$). On average, when computed on samples from the training dataset, the RMSE achieved by networks C+ and C− were 4.0$^{\circ }$ and 9.7$^{\circ }$ respectively. When computed on samples from the baseline dataset, the average prediction errors were 6.0$^{\circ }$ and 12.7$^{\circ }$ for networks C+ and C− respectively. These offline results show that adding contextual information to the input data allowed the context-aware network to reach significantly higher prediction accuracy than the context-unaware network. The outputs of statistical tests on the offline error are reported in Table 1.

**Table 1:** Output values from tests on offline network RMSE

	Training dataset	Baseline dataset
T	$-$ 10.69	$-$ 13.89
p	**2.047e**−**6**	**2.194e**−**7**

Significant differences are indicated by p values in bold

### Online performance metrics

A qualitative analysis of mean approach times reveals that condition C− elicited the longest approaches. Indeed, mean approach times in this condition were over 1.4 s for all subjects, whereas it was the case for only two subjects out of ten in condition C+, and none in condition N. This analysis also highlights that some subjects using hybrid control with network C+ managed to reach performance levels similar to those achieved with natural control. Overall, mean approach times were 0.82s, 1.22s and 2.31s for conditions N, C+ and C− respectively. The outputs of statistical tests on the two other online performance metrics are reported in Table 2.

**Table 2:** Output values from tests on online performance metrics

Metric	ANOVA/Kruskal–Wallis test	Paired tests—$\alpha _{corr} = 0.0167$			
			C+ vs C−	C+ vs N	C− vs N
AS	F = 41.36	T	7.846	− 4.834	− 11.61
	**p = 6.024e−9**	p	**2.585e**−**5**	**9.291e**−**4**	**1.022e**−**6**
SV	H = 20.11	W	0	2	0
	**p = 4.301e−5**	p	**0.005062**	**0.009344**	**0.005062**

Significant differences are indicated by p values in bold

**Fig. 4:**
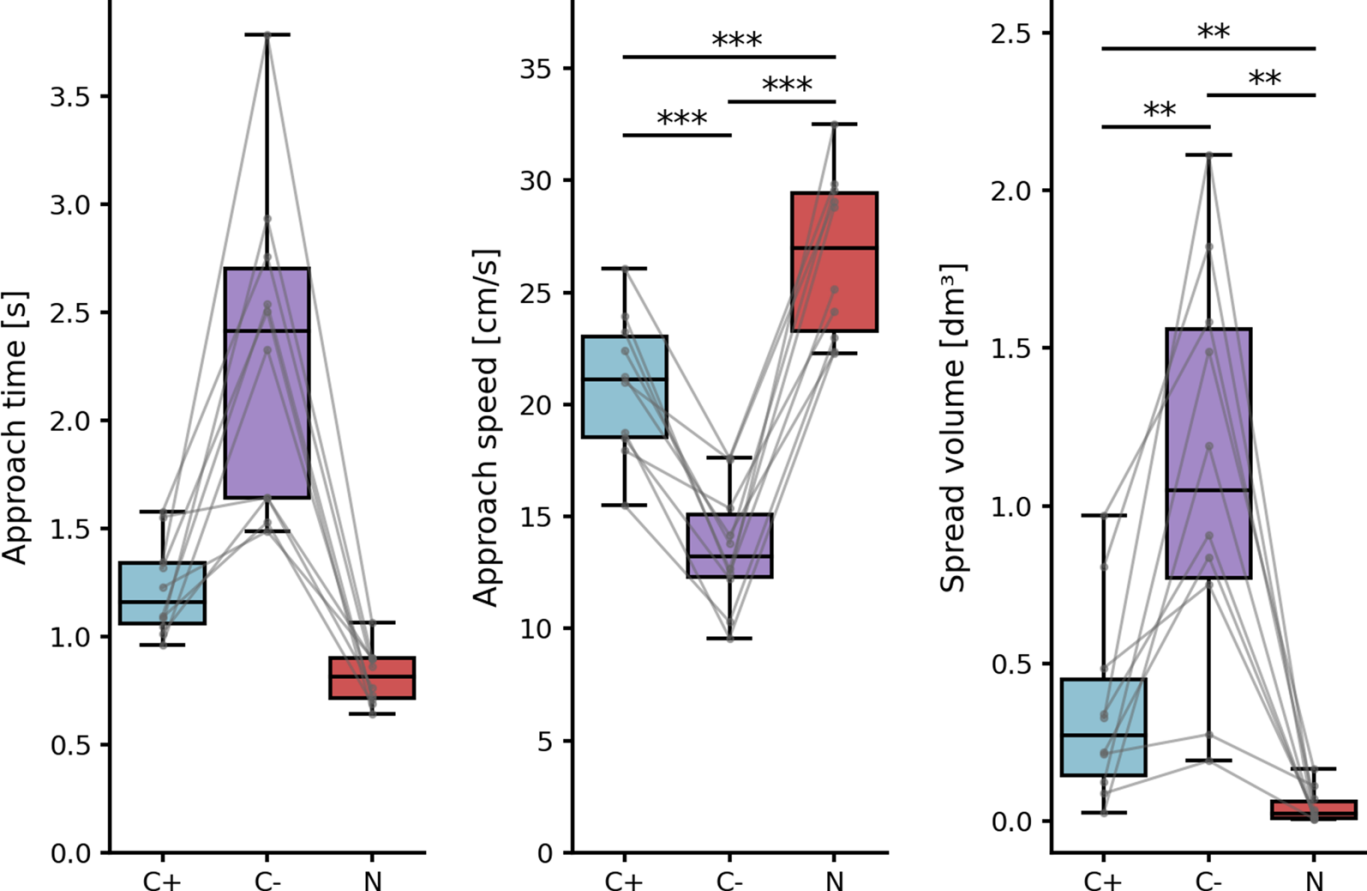
Results on performance metrics. Each grey line corresponds to a subject. Boxes show first and third quartiles, whiskers show min and max values. Left: approach time, lower is better. Center: approach speed, higher is better. Right: shoulder spread volume, lower is better. Blue: hybrid control with network C+; purple: hybrid control with network C−; red: natural control (N). Significant differences are indicated with stars: **$\hbox {p} < 0.01$; ***$\hbox {p} < 0.001$

Regarding approach speeds, results were significantly different for each condition ($\hbox {p} < 0.001$) and consistent with the pattern obtained with approach times (see Fig. 4). In particular, hybrid control with network C− elicited slower approach periods (mean AS 13.6 cm/s) than with network C+ (mean AS 20.9 cm/s). This suggests that the C− network offered poorer control than the C+ network during the task, resulting in subjects struggling more often to bring the virtual hand into the target zone. As expected, the fastest reaching was achieved with natural control (mean AS 26.7 cm/s).

Regarding shoulder position spread volume, the analysis revealed that the scattering of shoulder positions was significantly different for each condition ($\hbox {p} < 0.01$). Natural control (condition N) elicited very little shoulder motion from all subjects (mean SV 47 cm^3^, about a third the volume of a tennis ball), likely to correspond to the amount of scapular motion naturally involved in the reaching of target positions. Conversely, subjects performed much more shoulder motion when driving the virtual arm with network C− (mean SV 1.12 dm^3^, about the volume of seven tennis balls). This condition also displays high inter-subject variability: some subjects managed to achieve the task with only limited shoulder motion (min SV 0.19 dm^3^) whereas others had to resort to wide and/or frequent shoulder motion to complete the task (max SV 2.11 dm^3^). In such cases, shoulder motion exceeding the range of scapular motion implies that compensatory trunk motion was involved. For all subjects, hybrid control with network C+ elicited less shoulder position scattering (mean SV 0.36 dm^3^, about the volume of two tennis balls) than with network C−, sometimes as little as with natural control.

### Variability of reaching postures

**Fig. 5:**
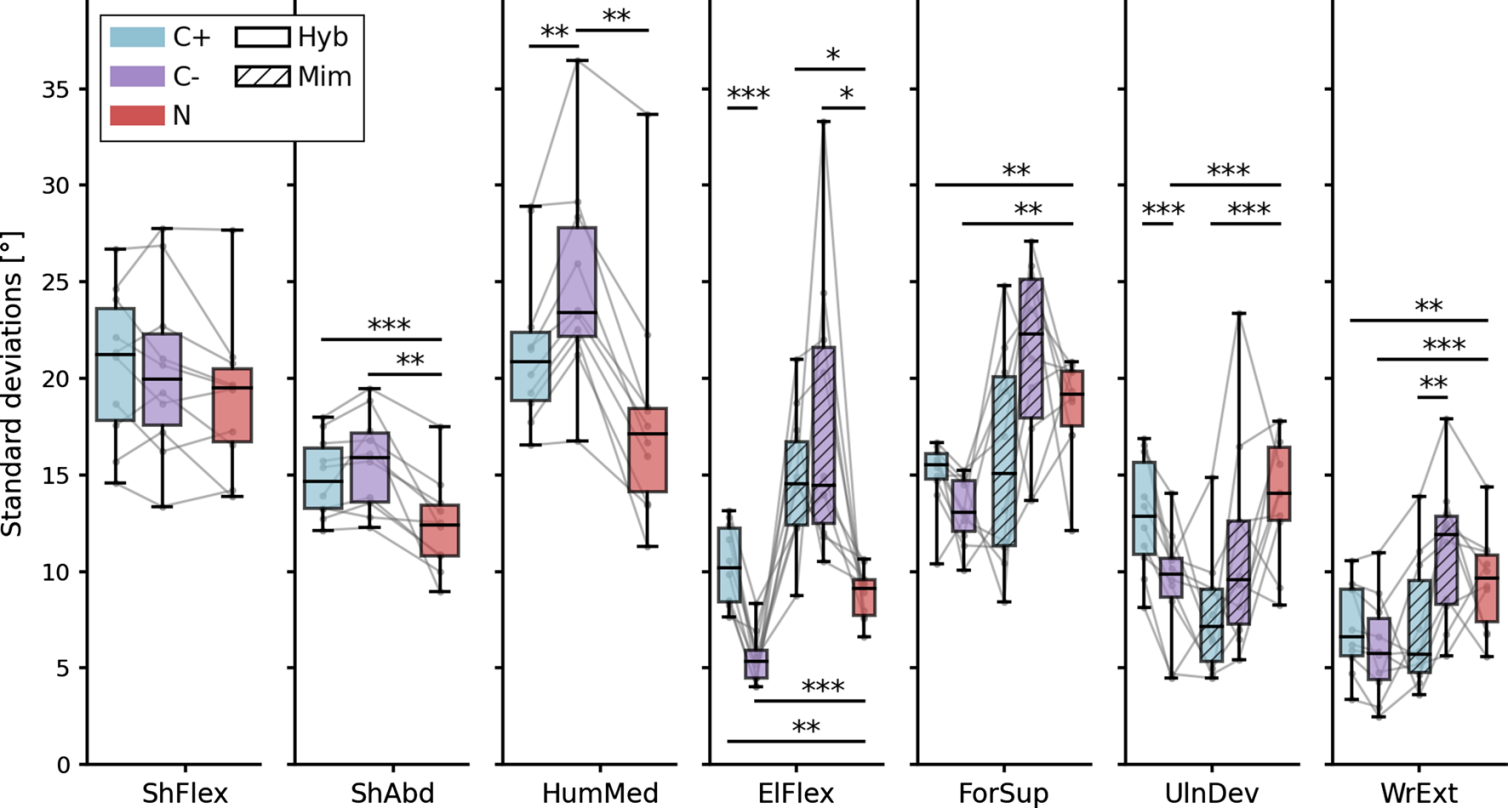
Results on joint angle standard deviations. Each grey line corresponds to a subject. Boxes show first and third quartiles, whiskers show min and max values. *ShFlex* shoulder flexion, *ShAbd* shoulder abduction, *HumMed* humeral lateral rotation, *ElFlex* elbow flexion, *ForSup* forearm supination, *UlnDev* ulnar deviation, *WrExt* wrist extension. Blue: condition C+; purple: condition C−; red: condition N. Solid: virtual arm actual behavior (C+, C− and N); hatch pattern: mimicked real arm motion (MC+ and MC−). Significant differences are indicated with stars: *$\hbox {p} < 0.0167$; **$\hbox {p} < 0.01$; ***$\hbox {p} < 0.001$

The outputs of statistical tests on the variability of reaching postures are reported in Table 3. The distributions of joint angles’ standard deviations are illustrated in Fig. 5.

**Table 3:** Output values from tests on joint angle variability

DoF	ANOVA/Kruskal–Wallis test	Paired tests—$\bf \alpha _{corr} = 0.0167$			
*Virtual arm actual behavior*		C+ vs C−		C+ vs N	C− vs N
ShFlex	F = 0.4457		N/A	N/A	N/A
	p = 0.6450				
ShAbd	F = 5.136	T	− 2.376	4.962	4.328
	p = **0.01287**	p	0.04153	**7.789e**−**4**	**0.001912**
HumMed	H = 9.185	W	0	8	0
	p = **0.01013**	p	**0.005062**	0.04685	**0.005062**
ElFlex	F = 23.26	T	7.442	3.390	− 5.236
	p = **1.342e−6**	p	**3.924e**−**5**	**0.007999**	**5.375e**−**4**
ForSup	H = 16.47	W	6	0	1
	p = **2.656e−4**	p	0.02842	**0.005062**	**0.00691**
UlnDev	F = 5.867	T	6.212	− 1.331	− 8.753
	p = **0.007657**	p	**1.567e**−**4**	0.2158	**1.071e**−**5**
WrExt	F = 4.772	T	2.446	− 4.166	− 6.056
	p = **0.007657**	p	0.03697	**0.002428**	**1.890e**−**4**
*Mimicked real arm motion*		MC+ vs C−		MC+ vs N	MC− vs N
ElFlex	H = 16.97	W	20	0	0
	p = **2.062e**−**4**	p	0.4446	**0.005062**	**0.005062**
ForSup	H = 6.227	W	9	17	17
	p = **0.04444**	p	0.05934	0.2845	0.2845
UlnDev	F = 5.593	T	− 2.525	− 4.980	− 1.492
	p = **0.009284**	p	0.03252	**7.595e-4**	0.1699
WrExt	F = 3.516	T	− 4.276	− 2.911	1.383
	p = **0.04394**	p	**0.002063**	0.01727	0.2001

Significant differences are indicated by p values in bold

*ShFlex* shoulder flexion, *ShAbd* shoulder abduction, *HumMed* humeral lateral rotation, *ElFlex* elbow flexion, *ForSup* forearm supination, *UlnDev* ulnar deviation, *WrExt* wrist extension

#### Virtual arm actual behavior

This part of the analysis focuses on the postures of the virtual arm that was driven by the subject and visible in the virtual environment during the experiment. For shoulder flexion, no difference between groups was revealed by this analysis (mean SDs around 20$^{\circ }$). Regarding shoulder abduction, the variability for group N (mean SD 12.4$^{\circ }$) was lower than from both other groups, C+ and C− (mean SDs $> 14.8^{\circ }$, $\hbox {p} < 0.002$), which were not different from each other. Humeral rotation angles in group C− (mean SD 24.9$^{\circ }$) were significantly more variable than in groups C+ and N (mean SDs $< 21.6^{\circ }$, $\hbox {p} < 0.006$) and a slight trend towards difference was found between groups C+ and N (mean SDs 21.6$^{\circ }$ and 18.1 respectively, p = 0.047).

Overall, this pattern of results on two out of three shoulder DoFs suggests that joint angles were more variable for hybrid controls C+ and C− when compared to N. The analysis also revealed a substantially higher variability for condition C− than for C+ on one of these DoFs. This is consistent with higher compensatory movements in condition C− than C+, as well as in both of these conditions when compared to N.

Regarding distal joints, elbow angle variability was significantly different between each pair of the set {N, C+, C−}. In particular, the angles predicted by network C+ were notably more variable than those predicted by network C− (mean SDs 10.3$^{\circ }$ vs 5.5$^{\circ }$, $\hbox {p} < 0.0001$). A similar difference between groups C+ and C− was found for ulnar deviation (mean SDs 12.9$^{\circ }$ vs 9.4$^{\circ }$, $\hbox {p} < 0.0002$) but not for forearm supination or wrist flexion. However, the analysis on these two later DoFs revealed that the angle variability was higher for natural control (mean SDs 18.6$^{\circ }$ and 9.4$^{\circ }$ respectively) than for both hybrid control strategies ($\hbox {mean SDs} < 15.1^{\circ }$ and $< 7.1^{\circ }$ respectively, $\hbox {p} < 0.007$).

Overall, these results suggest that the distal joint angles predicted by network C− display a certain lack of variability that may lead to poorer performance, as highlighted by the performance metrics. In particular, the elbow is a key joint whose wide angular range is critical to perform reaching across the whole workspace. In this context, too little variability on this joint angle may restrain the fraction of workspace reachable only with arm motion, therefore eliciting more compensatory trunk motion.

#### Mimicked real arm motion

This part of the analysis focuses on how well the subject’s real distal arm movements continued to follow the virtual arm’s movements generated by the neural network. Real elbow movements elicited under conditions C+ and C− were more variable than under condition N (groups MC+ and MC: mean SDs > 14.6$^{\circ }$, group N: mean SD 8.8$^{\circ }$, $\hbox {p} < 0.006$). The analysis revealed no significant difference for forearm supination. Conversely, ulnar deviation variability was higher for group N (mean SD 13.9$^{\circ }$) than for group MC+ (mean SD 7.8$^{\circ }$, $\hbox {p} < 0.0008$). Additionally, the only significant difference revealed for wrist extension was between MC+ and MC− (mean SDs 7.1$^{\circ }$ VS 11$^{\circ }$, $\hbox {p} < 0.003$).

### Joint angle synergies

#### Distribution of explained variance

**Fig. 6:**
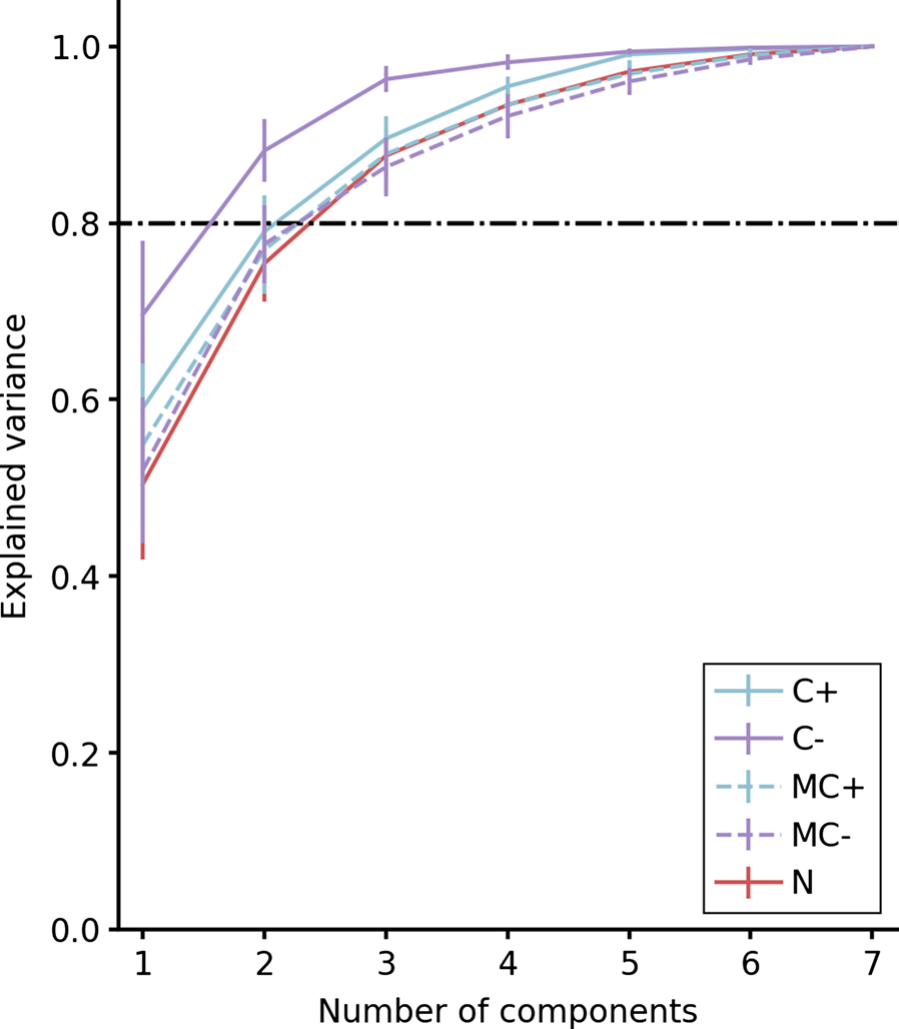
Cumulated ratios of explained variance against number of PCs, by group. Blue: condition C+; purple: condition C−; red: condition N. Solid line: hybrid control (groups C+ and C−); dashed line: natural control (groups MC+ and MC−). The dash-dotted line indicates 80% of explained variance

The first step in the analysis of joint angle synergies focused on the explained variance ratios associated with the first PCs, which represent the primary postural synergies. The higher this ratio is, the more prominent are the corresponding synergies in the posture data. We sorted the explained variance ratios by PC and group, and averaged over all subjects. The average cumulated ratios of explained variance are shown in Fig. 6.

This revealed that overall, PCs extracted from postures in group C− explained more variance than those in group C+. Indeed, cumulated ratios for two to four PCs were higher in group C− than in group C+ for all subjects. In particular, the amount of explained variance by the first three PCs reached an average of 96% in group C− whereas it remained under 90% in group C+. This is consistent with condition C− eliciting less differentiated control of individual distal arm angles, perhaps related to more reliance on compensatory shoulder motion.

#### Comparison of PC subspaces

The similarity between motor synergies was assessed using a measurement of geometric proximity between subspaces generated by PC vectors from two given PCAs. As detailed in the section “Analysis of joint angle synergies with PCA”, this proximity is measured using the minimal angle that rotates one subspace into the other. This method requires to choose *n* the number of PCs spanning these subspaces. To identify values of *n* for which such a subspace encompasses the primary joint angle synergies revealed by the PCA, we considered the cumulated ratios of explained variance. On one hand, at least three PCs are required to explain 80% of the variance or more for all groups. On another hand, using six or more PCs notably reduces the benefit offered by the PCA in terms of dimensionality reduction. Therefore, we chose to focus our analysis on values of *n* between 3 and 5.

This method was applied to perform two types of comparison: on one hand, between hybrid control and natural control in baseline phase (C+ versus N and C− versus N); on another hand, between hybrid control and the mimicked real arm motion produced in the same test phase (C+ versus MC+ and C− versus MC−). Using values of *n* ranging from 3 to 5, we computed the angular distances corresponding to these four comparisons for each subject, yielding four sets of ten angles for each value of *n*.

**Fig. 7:**
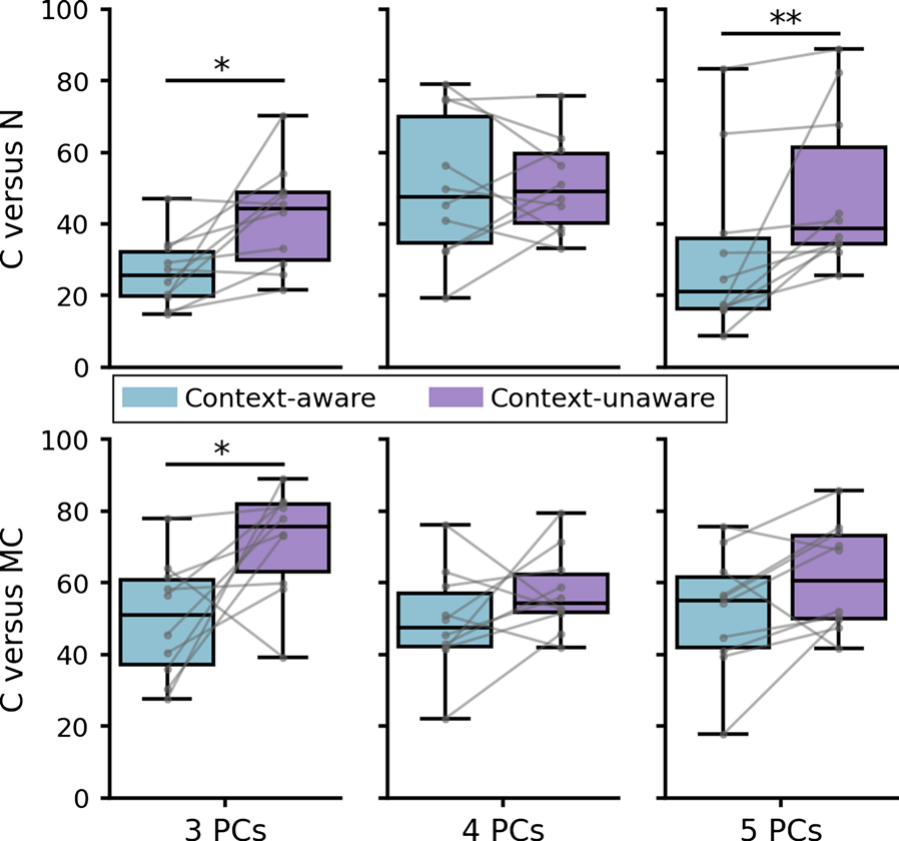
Results on angular distances between PC subspaces. Boxes show first and third quartiles, whiskers show min and max values. Top row: between hybrid controls and natural control in baseline phase. Bottom row: between hybrid controls and mimicked real arm motion produced in the same test phase. Blue: condition C+; purple: condition C−. Significant differences are indicated with stars: *$\hbox {p} < 0.05$; **$\hbox {p} < 0.01$

These sets of angular distances were compared two by two based on the type of comparison and number of PCs, using either paired T-tests or Wilcoxon tests depending on the normality of samples. Relevant statistical values from these tests are reported in Table 4 and results are shown in Fig. 7 in the form of boxplots.

**Table 4:** Output values from tests on distances between PC subspaces

	3 PCs	4 PCs	5 PCs
C versus N	T = − 3.020	T = − 0.075	W = 0
	p = **0.01449**	p = 0.9419	p = **0.005062**
C versus MC	T = − 2.727	T = − 1.390	T = − 2.058
	p = **0.02334**	p = 0.1979	p = 0.06975

Significant differences are indicated by p values in bold

Regarding the proximity with natural control in baseline phase, the analysis revealed that the angular distance between subspaces was significantly higher in condition C− when considering the first three or five PCs ($\hbox {p} < 0.015$). This result shows that the primary motor synergies emerging from hybrid control were closer to natural motor synergies in condition C+ than in condition C−. This is consistent with the differences in joint angle variability previously reported: SDs of angles predicted by network C+ were more often similar to those in natural control.

When comparing synergies between hybrid control and real arm motion in the same test phase, the only significant difference was found for subspaces based on three PCs (p = 0.023). Again, synergies in hybrid control were closer to those of the subject’s arm in condition C+ than in condition C− (mean distances 49.8$^{\circ }$ VS 71.7$^{\circ }$). The angular distance between subspaces spanned by five PCs seemed to display a similar trend, even though no significant difference was found (p = 0.070). Overall, these results suggest that the joint coordinations produced by network C+ are more similar to those underlying the subject’s natural arm motion.

## Discussion

We have explored the benefits of adding contextual information about the movement goal in the process of distal joint prediction for prosthesis control. We show that a context-aware network reconstructing four distal joints enables close to natural performance, with moderate compensatory movements from trunk and shoulder, for picking and placing a bottle in various positions and orientations. After discussing these results in relation to the literature, we identify remaining gaps and perspectives for real-world application for prosthetic control.

### Approach time and speed

Remarkably, average approach times performed using the context-aware network without any further training from the participants were close to natural (1.22 s as compared to 0.82 s), and much better than using a context-unaware network (2.31 s). Yet, approach speeds with control from the context-aware network were significantly lower than when the virtual arm was teleoperated by real arm movements in the natural baseline control conducted at the end of the experiment, which indicates that there is still room for improvements.

Out of the numerous studies that have explored proximal-to-distal joint predictions for prosthetic control [9–18], only one included the control of a sufficient number of distal joints to enable hand positioning to grasp objects with various positions and orientations [13]. Average movement times obtained in this study were much higher than here even after 10 days of practice (9.49 s and 5.76 s on the tenth session for the reconstructed and natural controls, respectively), but the task involved multiple components i.e. reach and grasp a bottle, bring it to the mouth, and release it to a given location. Yet, approach times for the first phase only (reach and grasp the bottle) remained between 4 and 6 s with the hybrid control, and around 3 s with natural movement control. Several reasons might explain the remaining difference with movement times we observed here.

First, we showed that using context-aware network greatly improves predictions as well as subject-in-the-loop performances. However, average approach times obtained with our context-unaware control were 2.31 s, which remains lower than the 4–6 s range in [13]. Importantly, an active grasp control was involved in [13], whereas in the present study the bottle was automatically grasped upon one second of consecutive holding within a strict tolerance to the goal position and orientation. Although this tolerance was somewhat relaxed in the test phase {4 cm, 10$^{\circ }$} as compared to that employed at initial acquisition {2 cm, 5$^{\circ }$}, it was still substantially lower than the {6 cm, 30$^{\circ }$} used in [13]. It remains that active grasp control is likely to have elicited longer movement times in [13] and will ultimately need to be included for real-world applications.

Additionally, [13] did not always use artificial networks trained on data from the same subject as the one operating the prediction-based control. In fact, subjects alternated sessions with a network trained on their own data versus a network trained on data from the particular control subject that elicited the worst predictions when applied on their own data. That way, subjects experienced controls that were tuned to them in both the best and worst possible manners. Remarkably, subjects performance were comparable for both cases by the end of the training sessions, which indicates that they were able to cope with a control based on other subjects’ data, as would necessarily be the case for people with upper-limb disability, from which obtaining baseline natural control data is not an option. Although this is promising for future applications of our context-aware strategy, we only used networks trained on data from the same subjects. Therefore, possible deteriorations when using data from other subjects remain to be evaluated.

Another difference in the present work relates to the restricted volume of the peripersonal space tested. Although our target arrangement spanned a sizeable proportion of space used during comfortable unconstrained reaches in front of the subjects, it is much smaller than the whole peripersonal space exploited in our daily activities. Furthermore, as soon as the object is in the hand, the subject must decide and command what to do with it. Although additional control features were included in [13] to bring the bottle to the mouth, efficient control strategies remain to be designed for the wide range of possible actions and peripersonal space, as well as efficient mechanisms to select the relevant context-aware strategy.

Yet another difference was that trunk movements were limited by elastic bands in [13], while discouraged but permitted in our case. This was designed to promote sufficient success rate to enable meaningful comparison on the different dependent variables. Indeed, it was evident from pilot testing that numerous targets would not have been reachable should we have restricted trunk movements. In the end, our choice was justified by the usefulness of subsequent analyses performed on joint coordination and compensatory movements associated with our experimental conditions.

### Joint coordinations and compensatory movements

Higher shoulder movements observed here in the context-unaware control, as compared to the context-aware control, were contingent upon lower variability of artificial movements for both the elbow and radial-ulnar deviations at the wrist. This suggests that wider shoulder motion could have been employed to compensate for shorter ranges of distal motion.

Postural synergies were found simpler with the context-unaware control, as indicated by more variance explained by the first few components when principal component analyses were conducted on joint coordination in the context-unaware condition, as compared to both the context-aware and the natural control conditions. In the absence of additional knowledge of the movement goal, predictions from the context-unaware network result in reduced distal joints motion that naturally required higher compensations from the trunk and shoulder to perform the task.

Postural synergies observed in the controlled joint coordination were also found to be more similar to those of natural movements with the context-aware than with the context-unaware control, as indicated by lower angular separation between subspaces spanned when considering the first few components that explained a sufficient amount of variance [8]. In addition to the overall higher performance obtained with lower compensatory movements, the closeness to natural movements associated with the context-aware control is potentially important. Indeed, we recently showed that human-likeness impacts robotic arm endpoint control, possibly through embodiment via increased sense of agency [30].

### Comparison to a model-based approach

In this experiment, the network C+ uses knowledge of proximal joint angles and target location to predict distal joint angles that bring the virtual hand on the target. In this context, one can consider that it implicitly performs some kind of inverse kinematics (IK) solving despite not being model-based. Alternatively, a common approach to IK solving would employ a mechanical model of the limb to compute the distal joint angles minimizing the distance between the target and the hand. However, when the upper arm posture would prevent the hand from reaching the target, such control is likely to result in the distal segments always pointing towards the target. As a result, these joints may seem to be completely out of the user’s control, which could be inappropriate in terms of sense of agency [30, 31]. Filtering or motion blending methods could be employed to limit the occurrence of such unexpected, target-driven distal motion, as opposed to subject-driven motion. Systematic evaluation of the performance and user acceptability of model-based approaches will be important for future works.

A recent study [22] proposed an alternative model-based control where IK solving is used in synergy with upper-arm motion. This control drives the distal segments so that the virtual hand’s position stays along the straight line joining the shoulder and target. As a result, the hand gets closer to the target as the subject brings the elbow forward. This “task space”-based control was tested in a simplified setup where the distal joint prediction is limited to the elbow and the reaching task only covers four targets placed in the same vertical orientation. The performance obtained with this control appeared to be on par with that of a simpler joint space synergistic control, suggesting that similar motor behaviors can be obtained using these two distinct approaches.

### Limits and perspectives for prosthetic control

Prosthetic simulations in virtual reality are useful research tools [13, 14, 18, 22, 24, 25] but real prostheses present additional challenges. Comparison with movement times obtained in related works such as [16, 30, 32] is informative, although obtained while reaching unoriented targets with simpler control strategies. In a study of upper-limb amputees equipped with a real prosthetic elbow whose joint velocity was controlled by shoulder velocity [16], subjects reached various target positions with an average movement time of 2.4 s, more than twice that produced by healthy subjects reaching the same targets with their own arm (1.1 s). Comparable reaches took on average 2.9 s when a robotic arm endpoint was teleoperated by subjects’ real arm movements in [30] and movement times increased to 4–5 s when a comparable robotic interface was controlled from isometric forces instead of real movements [32]. This large difference in movement times might be related to real-world mechatronic considerations. A simulated arm in a virtual reality setup can “instantaneously” move from one posture to the other, whereas robotic and prosthetic arms are limited by the strength and speed of their motors operating against gravity and inertia. Most prosthetic limbs have the additional problem of slippage between socket and stump, which will introduce errors and even instability. In this context, osseointegration is increasing the potential applicability of controls based on residual motion [16].

Although our results are promising for potential application to real-case scenarios in prosthesis control, several gaps remain. Among those, we already identified the necessity to include an active grasp control, mechatronic considerations to realize real movements absent in virtual reality testing, and subject-specific tuning of the context-aware strategy to meet individual requirements of people with upper-limb amputation. To these, we can add real testing on people with upper-limb amputation, and finding reliable solutions for automatic detection of contexts as well as their associated control strategies.

Combining kinematics and myoelectric signals [14, 33] could be used to integrate active grasp control to the present context-aware strategy. Most transradial prosthesis users are already familiar with myoelectric controls for hand opening and closing. Poor resolution and delays under visual control make it difficult to grasp fragile objects without crushing them, but this may be overcome by adding tactile sensing and feedback [34, 35]. To avoid the burden of noisy and hard to interpret myoelectric signals, and preserve advantages of kinematic-based control, [13] designed a proportional hand closing mechanism based on sternoclavicular protraction. Decoupling hand closure from this voluntary repositioning of the shoulder proved difficult in that particular instance [13] and appears as a general limitation of this strategy. With extended daily practice, real prosthesis users may learn strategies that escaped our normal volunteers and may be able to cope with coadaptation strategies in smart prostheses [18, 36]. The addition of vibrotactile feedback in lieu of proprioception [37] may also improve sensorimotor integration of the proposed control solutions.

In order to facilitate development and testing of prototype prostheses that will support different control schemes, it will be useful to take advantage of multiple robotic platforms that are increasingly accessible to researchers [38–40]. Among these, we recently proposed one that has the potential to attack most perspectives mentioned here [40]. The 3D-printed skeleton and control interface of this platform make it easy to reconfigure both from hardware and software perspectives. Furthermore, this platform is already interfaced with various relevant control signals (kinematics, myoelectric, gaze information) and control principles (inverse kinematics with both analytical and artificial networked-based solving), and now integrates cameras and prepackaged artificial intelligence solutions for efficient artificial network and computer vision implementations.

Technological simplicity, reliability and cosmesis are important personal considerations for prosthesis design and use [41–43]. The proposal here to add shoulder posture sensing plus gaze monitoring and video scene analysis will require substantial additional hardware:

- Complete shoulder and sterno-clavicular joint motion can be extracted from easily worn multiaxis “digital compass” sensors based on micro-electro-mechanical chips (MEMS, e.g. Honeywell HMC6343) affixed to the sternum, acromion and trans-humeral socket [13]. These can substitute for the reference-frame sensors used in the present experiment and most laboratories.
- Proof of principle for automatic object detection in [20, 21] were obtained with an expensive and somewhat heavy system that integrates camera and gaze tracking on glasses (Tobii Glasses 2), but lighter and more affordable options are starting to become available for augmented reality systems (e.g. Pupil Labs). To enable the proposed control scheme, the 3D position of the object detected in the field of view of the camera on eyeglasses would also need to be established in relation to both the current position and orientation of the head and the shoulder center of rotation. This could be provided by installing another digital compass chip on the eyeglasses.
- Extracting distance of an object is more difficult than its orientation and location in azimuth and elevation, but technology for extraction from binocular images or lidar reflection delay is rapidly improving. It is possible that the user’s visual perception of distance and kinematic control of reach extension would be sufficient without this aspect of machine vision.

Studies such as the one presented here represent demonstrations of technological feasibility. Clinical acceptance by patients will require benefits in activities of daily living that outweigh the inevitable increases in financial cost and donning/doffing time and system designs that achieve acceptable reliability and appearance. Fortunately, the required technology is likely to continue to improve in cost, size, simplicity and reliability. Effective industrial design will require selecting the most useful components and integrating them into attractive and cost-effective systems.

## Conclusion

The experiment reported in this paper shows that adding target-related contextual information can be beneficial to the control of an upper-limb prosthesis based on natural joint coordinations. Better reaching performance was achieved by a control strategy combining command signals from shoulder kinematics with information about target location and orientation such as can be obtained from gaze and scene analysis. Subjects using this combination achieved higher mean approach speeds as well as reduced compensatory motion compared to kinematics alone. Overall, the context-aware control strategy allowed subjects to achieve close to natural performance without training. Regarding applications in the field of prosthetics, these results are promising especially for the control of a multi-DoF prosthesis. However, notable challenges have yet to be overcome before such a control strategy can be implemented on a wearable, clinically relevant device.

## Data Availability

The datasets used and analysed during this study are available from the corresponding author on reasonable request.

## Abbreviations

3D: Three-dimensional
DoF: Degree of freedom
SD: Standard deviation
VR: Virtual reality
C+: Context-aware network
C−: Context-unaware network
MC+: Mimicked natural motion during condition C+
MC−: Mimicked natural motion during condition C−
RMSE: Root mean square error
SV: Shoulder position spread volume
AS: Approach speed
AT: Approach time
PCA: Principal components analysis
PC: Principal component
VS: Versus
ANOVA: Analysis of variance
ShFlex: Shoulder flexion
ShAbd: Shoulder abduction
HumMed: Humeral lateral rotation
ElFlex: Elbow flexion
ForSup: Forearm supination
UlnDev: Ulnar deviation
WrExt: Wrist extension
MEMS: Micro-electro-mechanical sensor
lidar: Light detection and ranging
